# Impact of Modified Diet, Swallowing Exercises, and Neuromuscular Electrostimulation on Severity of Oropharyngeal Dysphagia of Geriatric Patients

**DOI:** 10.3390/medicina60121927

**Published:** 2024-11-23

**Authors:** Margarita Rugaitienė, Vita Lesauskaitė, Ingrida Ulozienė, Gerda Kalinauskaitė, Marius Juška, Gytė Damulevičienė

**Affiliations:** 1 Clinical Department of Geriatrics, Lithuanian University of Health Sciences, 44307 Kaunas, Lithuaniamarius.juska@lsmu.lt (M.J.); gyte.damuleviciene@lsmu.lt (G.D.); 2 Department of Otorhinolaryngology, Lithuanian University of Health Sciences, 44307 Kaunas, Lithuania; ingrida.uloziene@lsmu.lt

**Keywords:** oropharyngeal dysphagia, aspiration risk, water swallow test, fiberoptic endoscopic evaluation of swallowing, neuromuscular electrical stimulation, swallowing exercises, modified diet

## Abstract

*Background and Objectives*: Oropharyngeal dysphagia is a common swallowing disorder, characterized by difficulties in moving food and liquids from the mouth to the esophagus; it is particularly prevalent among older adults with neurological conditions. This study aimed to evaluate the effectiveness of a short-term complex treatment protocol combining dietary modifications, swallowing exercises, and transcutaneous neuromuscular electrostimulation in reducing the oropharyngeal dysphagia severity and aspiration risk among geriatric patients. *Materials and Methods*: A total of 64 participants aged 60 and older, with oropharyngeal dysphagia, at LSMU Kaunas Hospital between May 2021 and April 2023, were included in the study after excluding those with significant comorbidities. Diagnostic assessments included the water swallow test and Fiberoptic Endoscopic Evaluation of Swallowing, conducted before and after treatment. *Results*: The results indicated a statistically significant reduction in the severity of oropharyngeal dysphagia, with 18.8% of patients showing improvements from moderate to mild dysphagia and 33.3% from severe to moderate. Additionally, the median PAS score was four points (IQR 3–6) before treatment and significantly decreased to three points (IQR 2–4) after treatment (*p* < 0.001). *Conclusions*: These findings suggest that even a short-term multidisciplinary approach that lasts 10 days can effectively alleviate the symptoms of oropharyngeal dysphagia, enhance patient safety, and improve swallowing among geriatric patients suffering from this condition.

## 1. Introduction

Oropharyngeal dysphagia (OD) has already been recognized as a geriatric syndrome because it fulfils all of the necessary criteria to be considered as such [1]. Oropharyngeal dysphagia can result from disorders affecting the oral preparatory phase and/or the pharynx [2]. Most often, OD occurs in people with various diseases, like Parkinson’s disease, dementia, multiple sclerosis, amyotrophic lateral sclerosis, stroke, pseudobulbar palsy, dermatomyositis, myasthenia gravis, and muscular dystrophy. Moreover, it might be a symptom of head and neck cancer or a complication due to radiation therapy or surgical treatment for this type of cancer [3,4,5].

OD in older adults causes serious health problems, significantly impacting their nutritional status, physical function, and lifespan. Ineffective swallowing results in malnutrition, dehydration, sarcopenia, weakened immunity, and poor wound healing, which increase the mortality risk. Unsafe swallowing causes aspiration pneumonia (AP), a major cause of death. OD also leads to anxiety and depression by impacting social interactions [1].

Oropharyngeal dysphagia is a fairly common clinical condition, affecting 13% of all people over 65 years of age. This percentage has been shown to increase with age, and the prevalence of OD among older hospitalized patients reaches 47% [3]. After a stroke, OD develops in 47–78% of patients [6,7].

OD can be diagnosed at the patient’s bedside using water swallow tests, but more reliable tests include the Fiberoptic Endoscopic Evaluation of Swallowing (FEES) and videofluoroscopy (VFS) [8]. The findings might be assessed using the Rosenbek Penetration–Aspiration Scale (PAS) [9].

The main clinical goal of OD treatment is symptom relief and the prevention of serious complications, such as aspiration pneumonia [10].

The use of pharmacological treatment for dysphagia is understudied, but the interest in this area continues to grow. Over the past few years, several studies have emerged demonstrating that transient receptor potential (TRP) channel agonists (capsaicin) reduce the latency of the swallowing response and the severity of dysphagia [11].

In a comparison of the currently available treatment strategies for OD, the best results are observed with rehabilitative swallowing muscle-strengthening measures and a modified diet [12]. To reduce the risk of liquid penetration into the respiratory tract, it is often recommended to change the viscosity of the liquid to different levels using a thickener [13].

Swallowing exercises improve swallowing function by enhancing swallow initiation, increasing the upward movement of the larynx, and decreasing the residues in the throat after swallowing [14].

Another very important part of complex OD treatment is neuromuscular electrostimulation (NMES). NMES is a procedure in which the swallowing muscles are stimulated for a certain period with relatively short electrical impulses, which stimulate the peripheral nerve, which then triggers muscle contractions [15]. NMES helps to decrease the pharyngeal transit time and swallowing response time to enhance laryngeal elevation; it also promotes vocal fold closure, increases the frequency of swallowing, and protects the airways [16].

This work demonstrates that even a relatively short and simple course of complex OD treatment in geriatric patients is useful in reducing the severity of OD and the risk of aspiration, thereby reducing dietary restrictions caused by a modified diet and improving well-being.

The primary outcome of this study was swallowing function as measured by the PAS, FEES, the Eating Assessment Tool (EAT)-10 questionnaire, the Dysphagia Handicap Index (DHI), and the Swallowing-Related Quality of Life Tool (SWALL-QoL/SWALL-CARE). The results of this study focused on improving the quality of life of geriatric patients have already been published in Medicina: https://doi.org/10.3390/medicina60071021.

This study aimed to evaluate the effectiveness of a short-term complex treatment protocol combining dietary modifications, swallowing exercises, and transcutaneous neuromuscular electrostimulation in reducing the severity of oropharyngeal dysphagia among geriatric patients.

## 2. Subjects and Study Design

This study comprised patients treated at the Geriatric Department of the Geriatric Centre and Department of Physical Medicine and Rehabilitation No. 2 between May 2021 and April 2023. Both departments belong to the Lithuanian University of Health Sciences Kaunas Hospital (City Hospital). After their first examination by a geriatrician, 75 patients with suspected oropharyngeal dysphagia (history of stroke, neurological degenerative diseases, complaints characteristic of swallowing disorders, suspicion of aspiration pneumonia) were invited to participate in this study. The inclusion criteria were suspected oropharyngeal dysphagia, an age of 60 and older, good mental capacity and an ability to participate in rehabilitation, language proficiency in Lithuanian, and the provision of a written consent form from the patient to participate in the study. The exclusion criteria were significant respiratory or heart failure (tachycardia (HR 100 bpm)), shortness of breath (respiratory rate 20 bpm and SpO2 90%), severe malnutrition (clear evidence of sarcopenia, cachexia, and exhaustion), the terminal stage of an oncological disease, acute stroke, cardiac pacemakers, and advanced dementia.

**Figure 1:**
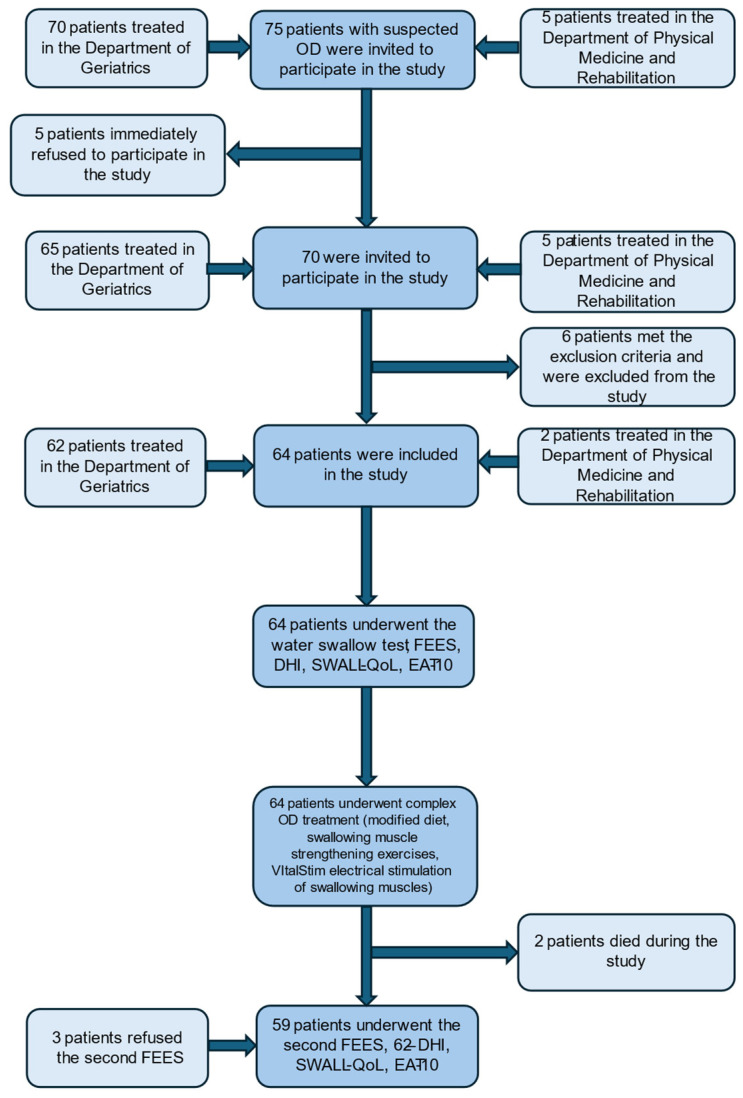
The study design.

Seventy-five patients with suspected oropharyngeal dysphagia were invited to participate in the study. Seventy patients were treated in the Geriatrics Department and 5 patients in the Physical Medicine and Rehabilitation Department. Five patients immediately refused to participate in the study or be investigated and treated for OD. Of the remaining 70 patients, 64 patients did not fulfil any of the exclusion criteria and were included in the study. Figure 1 demonstrates the study design.

Two patients were removed from the study after seven treatment sessions because they developed aspiration pneumonia and respiratory failure, ultimately leading to death. Their data were not included in the analysis of changes in the PAS and the severity of oropharyngeal dysphagia.

The study protocol was approved on 23 October 2020 by the Kaunas Regional Biomedical Research Ethics Committee, permit No. BE-2-12. The study was registered with ClinicalTrials.gov (#NCT05325658).

## 3. Materials and Methods

The water swallow test was administered to patients who were suspected of having oropharyngeal dysphagia [17]. This test was positive for all patients that were enrolled in this study.

A 3.7 mm high-definition video endoscope (STORZ, Tuttlingen, Germany) was used to perform endoscopic swallowing examinations, assessing the severity of dysphagia and the aspiration risk. The FEES evaluation was performed based on the FEES Examination Protocol [8]. Each patient underwent FEES twice: once before and once after receiving complex treatment.

The Penetration–Aspiration Scale (Rosenbek et al. 1996) was used to analyze the FEES results. The scores ranged from 1 (no penetration or aspiration) to 8 (aspiration), with scores of 2–5 indicating penetration (food reaching the vocal cords but not entering the airway) and scores of 6–8 indicating aspiration (food entering the airway) [18,19].

The aspiration risk was categorized as low (PAS 1–3), medium (PAS 4–5), or high (PAS 6–8) based on the Penetration–Aspiration Scale. The oropharyngeal dysphagia severity was classified as mild (minor swallowing problems correctable with diet changes), moderate (swallowing problems with some aspiration, correctable with diet changes), or severe (significant swallowing problems with clear aspiration, requiring artificial nutrition). The OD severity was described using criteria based on the FEES Examination Protocol (Table 1).

### 3.1. Treatment

This study’s treatment for oropharyngeal dysphagia in older adults combined the transcutaneous electrical stimulation of the swallowing muscles, dietary changes, and targeted exercises to strengthen the swallowing muscles.

During the study, transcutaneous electrical stimulation was performed by a certified healthcare professional (occupational therapist). The treatment used a VitalStim^®^ device (Chattanooga, Guildford, UK). A two-channel system with a current pulse frequency of 80 Hz and an impulse duration of 300 µs was used. Before the electrodes were placed on the neck skin, the anterior neck area was cleaned and disinfected, and all male subjects were shaved.

**Figure 2:**
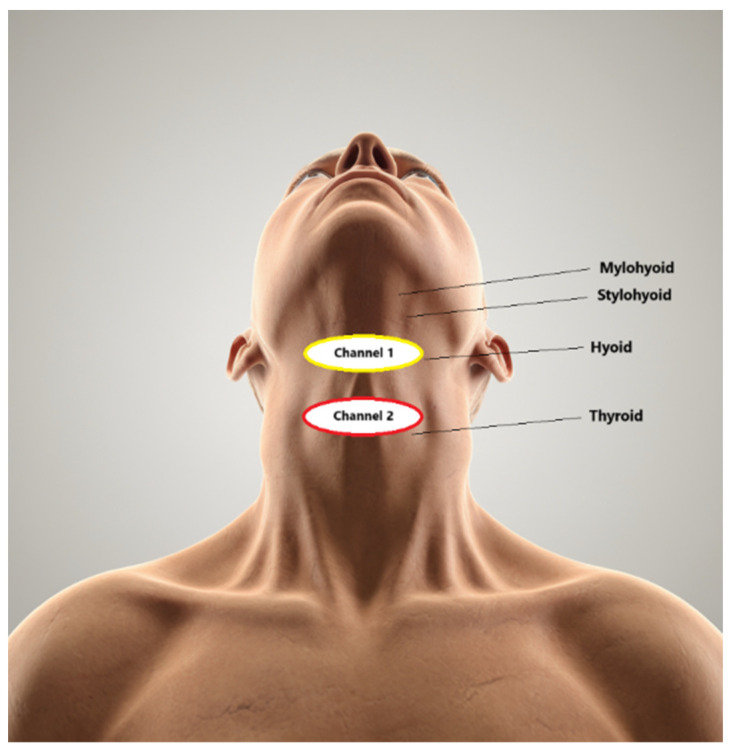
VitalStim channel placement used in the study.

Electrostimulation was established when a patient reported a tingling sensation. With the patient describing the sensation produced by the stimulation in detail, the amplitude was increased by 0.5 mA, starting at 0.5 mA, until the maximum level of tolerance was reached. During the stimulation, the patient, having felt a tingling sensation, had to sip a drink of the given thickness (perform swallowing movements). The duration of the procedure was 30 min. The placement of the electrodes (Figure 2) was selected based on the symptoms that caused the most discomfort to the patient, which included premature spillage, the early entry of the contents into the pharynx, residues at the base of the tongue, delayed initiation of swallowing, residues in the vallecula, and penetration/aspiration. The median NMES duration was 10 procedures.

Dietary adjustments were personalized based on each patient’s swallowing difficulties and aspiration risk, as determined by the endoscopic evaluation and the International Dysphagia Diet Standardization Initiative Committee (IDDSI) classification (Dublin, Ireland). Patients with mild dysphagia followed the Minced and Moist diet (MM5), while those with moderate to severe dysphagia received the Pureed diet (PU4). Two patients were prescribed enteral feeding. Fluids of the following thickness were recommended to the patients: level 4, extremely thick—all drinks, such as water, tea, coffee, and juice, were thickened to ~2858.70 mPa·s (50 s^−1^); level 3, moderately thick—all drinks were thickened to ~960.05 mPa·s (50 s^−1^)^2^); and level 2, mildly thick—all drinks were thickened to ~294.20 mPa·s (50 s^−1^). The viscosity for these levels of fluid was measured with an Anton Paar MCR 92 rheometer. A commercial medical thickener based on starch was used in this study [21].

A physiotherapist individually trained each patient in seven swallowing exercises (effortful swallow, tongue-hold swallow, supraglottic swallow, Shaker exercise, Mendelsohn maneuver, effortful pitch glide, and chin tuck) for approximately 20–30 min daily. The exercise program was standardized across all participants. The exercises and their descriptions are presented in Table 2.

The water swallow test and FEES were performed by the main investigator (physician, pulmonologist) and supervisor (experienced geriatrician) for all patients. Electrostimulation was performed by the same occupational therapist for every patient, and the swallowing exercises were taught and performed by the same physiotherapist.

### 3.2. Statistical Analysis

The statistical analysis was conducted using the SPSS 28.0 software (IBM Corp., released 2021; IBM SPSS Statistics for Windows (version 22H2); Version 28.0; Armonk, NY, USA). In the descriptive analysis, for continuous indicators, the mean ± standard deviation (SD) was calculated, and, for categorical variables, the absolute prevalence (n) and percentages (%) were used. In the inferential analysis, the relationships between categorical variables were assessed using the chi-squared test with Z-values, and the associations among continuous variables using the Pearson and Spearman correlation coefficients and the Mann–Whitney U test. The difference between two paired data was assessed using the Wilcoxon signed rank test. The level of statistical significance was set at *p* < 0.05.

## 4. Results

**Figure 3:**
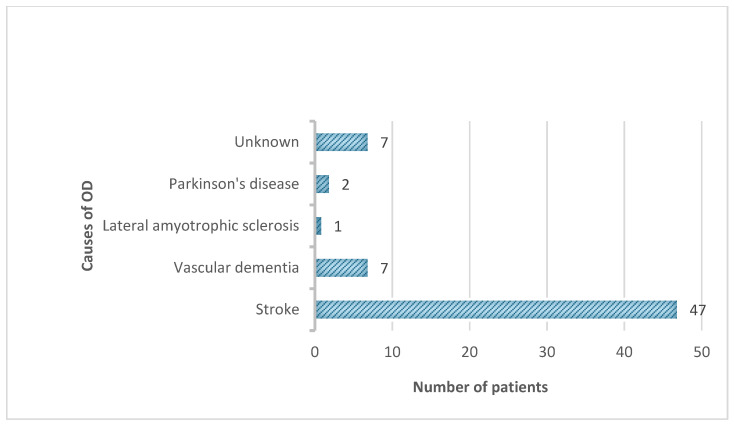
Causes of OD among study patients.

The average age of the participants was 77.8 years (with a standard deviation of 9.1 years), and 56.3% were female. The patients spent an average of 11.7 days in the hospital, and the duration of complex treatment was 9.33 (1.43) days (median—10 days). These calculations did not include one younger patient, who was treated under exceptional conditions for as long as 35 days. The oropharyngeal dysphagia of the patients participating in this study was mostly caused by neurological factors. A previous ischemic stroke was the most common reason. Figure 3 demonstrates the causes of OD among the study patients.

At the beginning of the treatment, the median electrical impulse level was 7 mA, and the mean was 5.49 (2.25) mA; during the treatment, it was increased to 7.04 (2.94) mA, while the median remained at 7 mA. The stimulus limit increased from 0.5–10.5 to 2.0–16.0 mA.

**Figure 4:**
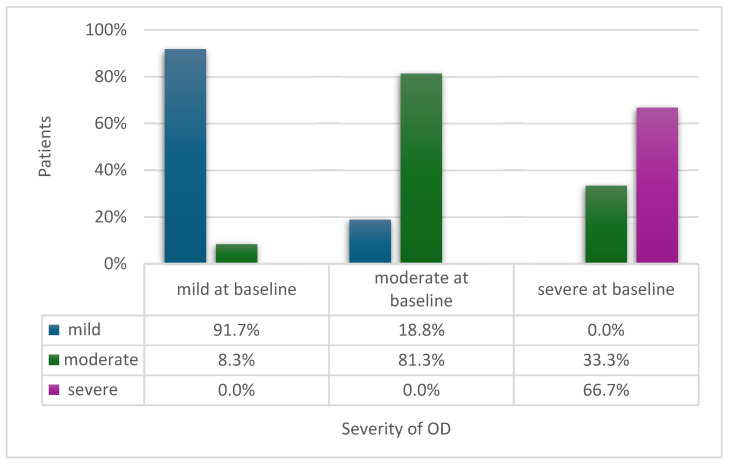
Changes in severity of oropharyngeal dysphagia after complex treatment (3 patients did not undergo FEES the second time (after treatment), the degree of severity remained primary, and 2 patients died during the study).

Initially, 18.8% of the patients had mild oropharyngeal dysphagia, 51.6% had moderate dysphagia, and 29.7% had severe dysphagia. The OD severity significantly changed after treatment (*p* < 0.002) [21]. One-third of the subjects diagnosed with severe OD were assessed as moderate after complex treatment, and 18.8% of subjects diagnosed with moderate OD were assessed as mild after OD treatment. The changes in the severity of dysphagia after treatment are shown in Figure 4.

The median PAS score was four points (IQR 3–6) before treatment and significantly decreased to three points (IQR 2–4) after treatment (Wilcoxon z = −4.08, *p* < 0.001) [20]. The PAS score in 23.7% of patients decreased by two points; in 15.3%, it decreased by one point.

**Figure 5:**
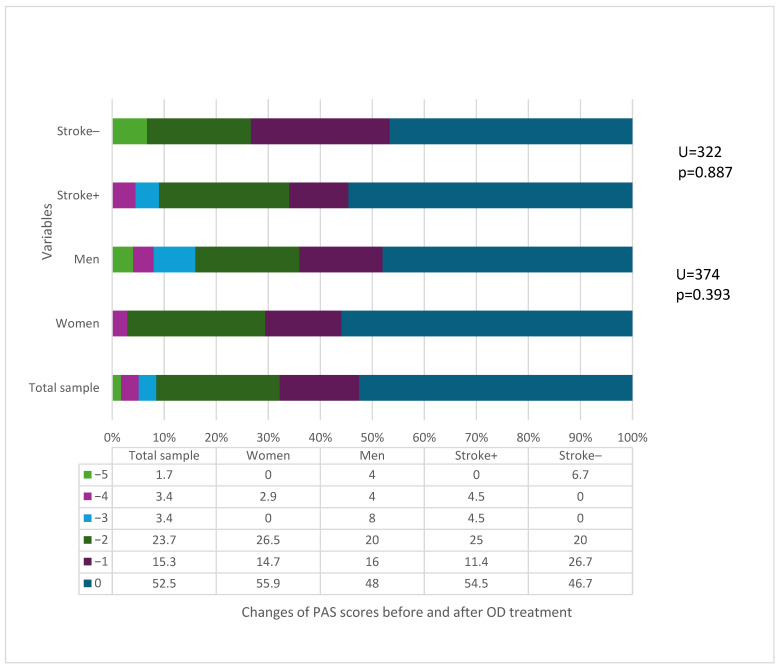
Interrelation among differences in Penetration–Aspiration Scale (PAS) scores before and after complex OD treatment and gender and cause of OD (3 patients did not undergo FEES the second time (after treatment), the severity level remained at the original one, and 2 patients died during the study; Mann–Whitney U test).

Changes in the PAS score following treatment were not significantly associated with the patient’s sex or the underlying cause of oropharyngeal dysphagia. This is shown in Figure 5. Moreover, changes in the PAS score were not associated with the patient’s age (*p* > 0.05).

**Figure 6:**
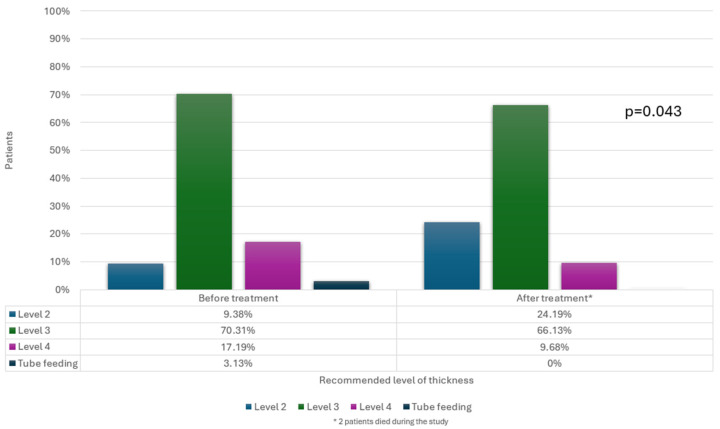
Changes in recommended level of fluid thickness before and after treatment.

This study found no significant association between changes in the OD severity after treatment and the age, sex, underlying diagnosis, duration of electrical stimulation, or stimulation intensity (*p* > 0.05). However, a significant association (*p* = 0.043) was found between the OD severity improvement and a reduction in the recommended fluid thickness. Specifically, 12 patients experienced both reduced OD severity and a decrease in the fluid thickness recommendation; two of these patients transitioned from tube feeding to an oral diet. Figure 6 illustrates these changes in the recommended fluid thickness.

Moreover, after the complex OD treatment, there was a statistically significant change in the modified diet given to the patients (Table 3).

## 5. Discussion

According to the literature, two diagnostic methods for OD have been competing for some time—videofluoroscopy and the endoscopic evaluation of swallowing. Authors often compare studies to discern and justify the superiority of one method. As endoscopic swallowing assessment and videofluoroscopy correlate with each other in the assessment of penetration, aspiration, and residues, they can be considered the gold standard for OD diagnosis [23]. The procedure also requires trained specialists to accurately interpret the results. The FEES procedure does not use radiation, so the test can be performed as many times as needed. In addition, this test provides immediate visual feedback, allowing for the assessment and diagnosis of OD immediately during the procedure. The equipment used for this examination is portable, so the procedure can be performed in a variety of settings, including examinations at the patient’s bedside, in outpatient clinics, or even at the patient‘s home. FEES is generally less expensive compared to VFS. FEES requires only local anesthesia in one nostril and is well tolerated by most patients, including children and those with severe dysphagia. As it requires skill and experience to perform and interpret the test, this test is only available in specialized centers [24,25].

Our study took place in a specialized geriatric center. After evaluating all aspects of the above-mentioned studies and considering the fact that geriatric patients would be studied, the FEES methodology was chosen for this study. Most patients tolerated the procedure well, a few patients tolerated it satisfactorily, and three patients refused re-examination, but no serious adverse events occurred.

After a diagnosis of OD, the first intervention is food and fluid modification to ensure safe and adequate nutrition for the patient. A changed fluid texture often causes negative responses in patients, so they avoid thickening their fluids. According to the literature, approximately 45% of patients with OD refuse to use thickeners, and 15% of these patients have a fatal outcome [26]. International experts state that fluid thickening, regardless of the severity of OD, helps to prevent complications caused by OD [27]. Baixauli and colleagues (2023) compared six commercial thickeners to identify the most palatable and comfortable option for patients [28]. The results indicated a preference for high-viscosity, smooth, tasteless thickeners derived from gum or soluble starch, avoiding those with noticeable particles. Starch-based thickeners have been described as having a gritty texture, intense starchy flavor, and unpleasant aftertaste. Studies have also shown that flavored thickeners bring improved sensory perceptions and, therefore, increased fluid intake [29]. Our study used a thickener based on starch. Some of the patients in this study had difficulty in adjusting to the new texture of the liquids that they drank. The severity of the oropharyngeal dysphagia in the patients who participated in this study after the treatment was linked to the consumption of thin fluids. As their OD symptoms improved, 12 patients were able to consume thinner fluids.

Exercises to strengthen the swallowing muscles are another very important part of the complex treatment of OD; the effectiveness of these exercises is described in many articles. Langmore and colleagues emphasized the importance of well-designed studies to prove that rehabilitation treatments work, as each patient is unique and factors such as age and health status can influence treatment outcomes [30]. Various factors can interfere with participation in rehabilitation following a stroke. Cognitive and emotional challenges often arise as consequences of a stroke, which can impede a person’s ability to comprehend or remember instructions or independently manage their rehabilitation plan [31]. The patients in our study were in sufficient physical and cognitive condition; they understood the instructions and followed them, so we had the opportunity to apply physical exercises that strengthened the swallowing muscles. On the other hand, the similarity of the patients was a limitation of this study, since the treatment methodology was applied to a narrower group of patients. In our study, the specific effect of swallowing muscle-strengthening exercises could not be assessed, as this method was part of the complex OD treatment. Balou and colleagues, in their study (2019), assessed the PAS before and after treating OD with swallowing muscle-strengthening exercises alone. The analysis showed that the swallowing safety did not improve statistically significantly after treatment, despite a median decrease in the PAS from 3 (unsafe) to 1 (safe/normal) after treatment [14].

According to a systematic literature review (Speyer, 2022), NMES was more effective than pharyngeal electrical stimulation (PES) in the treatment of OD. Positive results were obtained in 11 studies using NMES and only five studies using PES [16]. Most studies included in the meta-analysis involved stroke patients (31 studies); other diagnoses were Parkinson’s disease, cerebral palsy, and head and neck cancer. Across the 42 studies, VFS was mostly used to confirm the OD diagnosis, whereas six studies used FEES. NMES (muscular or sensory-level stimulation) was applied for 30 to 60 min and for 5 days a week; the treatment lasted 2 to 5 weeks. The VitalStim protocol was often used to stimulate the suprahyoid or suprahyoid and infrahyoid muscles, with stimulation at 80 Hz and greater according to the individual patient’s tolerance.

According to a literature review (Assoratgoon, 2022), the combination of sensory-level neurostimulation with conventional treatment (exercises that strengthen the swallowing muscles, modifications of the patient’s position during feeding, and diet changes) significantly improved the results compared to conventional treatment alone [6]. However, the results of the mentioned study were not related to the locations of the electrodes. The Intelect VitalStim^®^ device (Chattanooga Group, Hixson, TN, USA) was used in most cases as the stimulation device; other devices were the Stimplus^®^ (Cyber-medic Corp., Iksan, Republic of Korea), vocaSTIM-Master (Physiomed Elektromedizin, Schnaittach, Germany) and Gentle Stim^®^ (J Craft, Osaka, Japan).

In our study, the electrical stimulation of the swallowing muscles was applied in a similar mode, but the treatment lasted about 2 weeks. The strength of the electrical stimulation of the swallowing muscles increased during the stimulation, but the median did not change. Initially, for our patients, the sensory threshold was determined as the lowest current level; then, the current level was gradually increased. However, a higher level of stimulation could not be achieved, and the median remained at 7 mA, although the stimulus limit increased to 2.0–16.0 mA, so the stimulation was more sensory-level. According to the literature, both sensory and motor stimulation are beneficial for patients, especially for those who have experienced a stroke [32].

Thus, the results of this study show that, to reduce the severity of OD and the aspiration risk in geriatric patients, it is sufficient to apply a complex OD treatment including electrical muscle stimulation, physical exercises to strengthen the swallowing muscles, and a modified diet for at least 10 days.

## 6. Strengths and Limitations of the Study

This study has several strengths. Firstly, the same investigator performed all FEES assessments, and the electrical stimulation and exercises were administered by the same trained professionals. This reduced the inter-rater variability and strengthened the reliability of the findings. Secondly, the focus on geriatric patients within a specialized geriatric center adds clinical relevance, as this demographic often faces unique challenges in terms of swallowing disorders. These strengths contribute to the study’s reliability, enhancing the significance of its findings in understanding and managing OD in older adults.

This study also has several limitations that should be considered when interpreting the findings. First, the long-term effectiveness of the interventions remains unknown. The observed improvements may not be maintained over time without ongoing treatment, suggesting the need for further research that extends to home-based care. Furthermore, participants with significant comorbidities were excluded from the study, which may restrict the applicability of the findings to the broader geriatric population, who often face multiple health challenges. As a result, the complex treatment was tailored to motivated patients who could perform swallowing exercises and actively participate in neuromuscular stimulation, making it unclear whether this approach would be beneficial for individuals with more severe cases of oropharyngeal dysphagia. Additionally, the real function of the pharynx was not assessed with specific techniques (e.g., pharyngeal manometry), but this might be relevant to the swallowing function after the complex treatment is applied. Lastly, the participants’ dysphagia had various causes, which could have influenced the consistency of the treatment responses. Future studies should consider grouping the patients according to the specific conditions that lead to dysphagia for a more targeted treatment approach.

These limitations help to frame the results and indicate areas for future research, highlighting the importance of understanding the broader context of oropharyngeal dysphagia treatment in geriatric patients.

## 7. Conclusions

Even a short-term, 10-day (once a day, 5 days per week, for 2 weeks) complex OD treatment, in which geriatric patients are given a modified diet, seven 20–30-min-long swallowing muscle-stimulating exercises, and 30–40-min-long transcutaneous neuromuscular electrostimulation, can reduce the severity of oropharyngeal dysphagia. At the same time, it can reduce the recommended level of fluid thickness and the restrictions imposed by a modified diet.

## Figures and Tables

**Table 1:** Description of oropharyngeal dysphagia degrees of severity based on FEES.

Degree of Severity of Oropharyngeal Dysphagia	
Mild	Signs during the examination: ⁻ a small amount of saliva accumulated; ⁻ none or trace residue in vallecula/pyriform sinus (0–1 points in YPRSRS); ⁻ a slightly impaired formation of “white out”; ⁻ sensitivity (evaluated by touch test) is sufficient or slightly impaired—the edge of the epiglottis or the area around it is sensitive; ⁻ sufficient or slightly impaired retraction of the base of the tongue; ⁻ a 15 mL drink portion is tolerated; ⁻ repeated swallowing movements may be required; ⁻ a drink of I–II thickness is safe to swallow.
Moderate	Signs during the examination: ⁻ significantly increased amount of saliva (residue in the piriform sinuses, on the arytenoids); ⁻ mild–moderate residue amount in vallecula/pyriform sinus (2–3 points in YPRSRS); ⁻ very weak or no “white out” is observed; ⁻ swallowing movements are delayed; ⁻ impaired sensitivity—sensitive edge/middle of the epiglottis; ⁻ signs of aspiration when drinking water and a drink of I degree of thickness; ⁻ incomplete closure of the epiglottis; ⁻ moderate retraction of the base of the tongue; ⁻ a small portion of a drink (10–15 mL) is tolerated; ⁻ several repeated swallowing movements are required (two–three); ⁻ a drink of II–III degrees of thickness is swallowed the best.
Severe	Signs during the examination: ⁻ before drinking liquids, abundant residue of saliva and other liquids/food can be seen in the glottis, in the piriform sinuses, near the glottis (propulsion deficit); ⁻ severe residue amount in vallecula/pyriform sinus (4 points in YPRSRS); ⁻ there is no “white out”; ⁻ liquids consumed involuntarily run towards the glottis (posterior oral incontinence); ⁻ swallowing movements occur late (delayed pharyngeal phase); ⁻ sensitivity is very impaired—only the base of the epiglottis is sensitive or it is not sensitive at all; ⁻ signs of aspiration are observed during the examination; ⁻ possible regurgitation of the swallowed drink; ⁻ no closure of the epiglottis; ⁻ several repeated swallowing movements are required (two–three); ⁻ a very small portion of a drink (5–10 mL) is tolerated; ⁻ a drink of III degree of thickness is swallowed the best.

YPRSRS—the Yale Pharyngeal Residue Severity Rating Scale, 0–4-point ordinal rating scale [20].

**Table 2:** Swallowing exercises used in the study [14,22].

Exercise	Expected Result	Instruction for the Patient
Effortful swallow	Enhance the activation of the pharyngeal constrictor muscles and the tongue base.	Raise your tongue to the hard palate. Swallow (do the act of swallowing) with all your effort. Imagine trying to swallow a golf ball.
Tongue-hold swallow	Enhance contraction of the superior pharyngeal constrictor muscle.	Keep your tongue gently bitten between your teeth. Try to swallow saliva with the tongue in this position.
Supraglottic swallow	Intentional closing of the laryngeal entrance.	Hold your breath. Try to swallow saliva. Cough.
Shaker exercise	Strengthening of hyolaryngeal elevation muscles.	Lie on your back. Raise your head and look at the tips of your fingers (do not raise your shoulders). Hold your head like this for 1 s and return to the starting position.
Mendelsohn maneuver	Intentionally extending the lifting of the hyoid and the larynx and the opening of the upper esophagus.	Begin the act of swallowing. When you feel that the larynx moves up, squeeze/contract the muscles. Stay in this position for 5 s. Relax and complete the act of swallowing.
Effortful pitch glide	Strengthening the long pharyngeal muscles and shortening the pharynx.	Take a deep breath. Say the sound “EEE” while changing the timbre of the voice.
Chin tuck	Strengthen the infrahyoid and the suprahyoid.	Sit on a chair. Place a ball under your chin. Bend your chin down to squeeze a (30.0 cm) inflatable rubber ball as much as you can.

**Table 3:** Modified diet prevalence and variations used in the study.

Modified Diet	Number of Patients Before Treatment	Number of Patients After Treatment	*p*-Value
MM5	16	24	<0.001
PU4	46	38	<0.001
Enteral feeding	2	0	<0.001
	64	62 *	

* 2 patients died during the study.

## Data Availability

All data are available from the corresponding author upon reasonable request.

## References

[1] Baijens L.W., Clavé P., Cras P., Ekberg O., Forster A., Kolb G.F., Leners J.C., Masiero S., Mateos-Nozal J., Ortega O. (2016). European society for swallowing disorders—European union geriatric medicine society white paper: Oropharyngeal dysphagia as a geriatric syndrome. Clin. Interv. Aging.

[2] Lembo A.J. (2022). Oropharyngeal dysphagia: Etiology and pathogenesis. https://www.uptodate.com/contents/oropharyngeal-dysphagia-etiology-and-pathogenesis.

[3] Petrović-Lazić M., Babac S., Ilić-Savić I. (2022). Oropharyngeal dysphagia in elderly persons: Etiology, pathophysiology and symptomatology. Sanamed.

[4] Lembo A.J. (2023). Oropharyngeal dysphagia: Clinical features, diagnosis, and management. https://www.uptodate.com/contents/oropharyngeal-dysphagia-clinical-features-diagnosis-and-management.

[5] Huynh T.T.M., Dale E., Falk R.S., Hellebust T.P., Astrup G.L., Malinen E., Edin N.F.J., Bjordal K., Herlofson B.B., Kiserud C.E. (2024). Radiation-induced long-term dysphagia in survivors of head and neck cancer and association with dose-volume parameters. Radiother. Oncol..

[6] Assoratgoon I., Shiraishi N., Tagaino R., Ogawa T., Sasaki K. (2023). Sensory neuromuscular electrical stimulation for dysphagia rehabilitation: A literature review. J. Oral Rehabil..

[7] Liang Y., Lin J., Wang H., Li S., Chen F., Chen L., Li L. (2021). Evaluating the efficacy of vitalstim electrical stimulation combined with swallowing function training for treating dysphagia following an acute stroke. Clinics.

[8] Hey C., Pluschinski P., Stanschus S., Euler H.A., Sader R.A., Langmore S., Neumann N. (2011). A documentation system to save time and ensure proper application of the fiberoptic endoscopic evaluation of swallowing (FEES^®^). Folia Phoniatr. Logop..

[9] Thiem U., Jäger M., Stege H., Wirth R. (2023). Diagnostic accuracy of the ‘Dysphagia Screening Tool for Geriatric Patients’ (DSTG) compared to Flexible Endoscopic Evaluation of Swallowing (FEES) for assessing dysphagia in hospitalized geriatric patients—A diagnostic study. BMC Geriatr..

[10] Nakato R., Manabe N., Hanayama K., Kusunoki H., Hata J., Haruma K. (2020). Diagnosis and treatments for oropharyngeal dysphagia: Effects of capsaicin evaluated by newly developed ultrasonographic method. J. Smooth Muscle Res..

[11] Cheng I., Hamad A., Sasegbon A., Hamdy S. (2022). Advances in the Treatment of Dysphagia in Neurological Disorders: A Review of Current Evidence and Future Considerations. Neuropsychiatr. Dis. Treat..

[12] Speyer R., Cordier R., Sutt A.L., Remijn L., Heijnen B.J., Balaguer M., Pommée T., McInerney M., Bergström L. (2022). Behavioural Interventions in People with Oropharyngeal Dysphagia: A Systematic Review and Meta-Analysis of Randomised Clinical Trials. J. Clin. Med..

[13] Hansen T., Beck A.M., Kjaesrgaard A., Poulsen I. (2022). Second update of a systematic review and evidence-based recommendations on texture modified foods and thickened liquids for adults (above 17 years) with oropharyngeal dysphagia. Clin. Nutr. ESPEN.

[14] Balou M., Herzberg E.G., Kamelhar D., Molfenter S.M. (2019). An intensive swallowing exercise protocol for improving swallowing physiology in older adults with radiographically confirmed dysphagia. Clin. Interv. Aging.

[15] Park J.S., Hwang N.K., Kim H.H., Lee G., Jung Y.J. (2019). Effect of neuromuscular electrical stimulation combined with effortful swallowing using electromyographic biofeedback on oropharyngeal swallowing function in stroke patients with dysphagia: A pilot study. Medicine.

[16] Speyer R., Sutt A.L., Bergström L., Hamdy S., Pommée T., Balaguer M., Kaale A., Cordier R. (2022). Neurostimulation in People with Oropharyngeal Dysphagia: A Systematic Review and Meta-Analysis of Randomised Controlled Trials-Part II: Brain Neurostimulation. J. Clin. Med..

[17] Kubota T., Mishima H., Hanada M. (1982). Paralytic dysphagia in cerebrovascular disorder–screening tests and their clinical application. Gen. Rehabil..

[18] Wirth R., Dziewas R., Beck A.M., Clavé P., Hamdy S., Heppner H.J., Langmore S., Leischker A.H., Martino R., Pluschinski P. (2016). Oropharyngeal dysphagia in older persons—From pathophysiology to adequate intervention: A review and summary of an international expert meeting. Clin. Interv. Aging.

[19] Rosenbek J.C., Robbins J.A., Roecker E.B., Coyle J.L., Wood J.L. (1996). A Penetration-Aspiration Scale. Dysphagia.

[20] Neubauer P.D., Rademaker A.W., Leder S.B. (2015). The Yale Pharyngeal Residue Severity Rating Scale: An Anatomically Defined and Image-Based Tool. Dysphagia.

[21] Rugaitienė M., Lesauskaitė V., Ulozienė I., Smičius L., Damulevičienė G. (2024). Impact of Modified Diet, Swallowing Exercises, and Electrostimulation on Quality of Life of Older Patients Suffering from Oropharyngeal Dysphagia. Medicina.

[22] Dehaghani S.E., Bakhtiyari J., Salmani M., Shahabi S., Tahooneh A., Alibakhshi H. (2023). Effects of Rehabilitative Exercises on Swallowing Function in Elderly People: A Pilot Randomized Controlled Trial. Middle East J. Rehabil. Health Stud..

[23] Labeit B., Ahring S., Boehmer M., Sporns P., Sauer S., Claus I., Roderigo M., Suntrup-Krueger S., Dziewas R., Warnecke T. (2022). Comparison of Simultaneous Swallowing Endoscopy and Videofluoroscopy in Neurogenic Dysphagia. J. Am. Med. Dir. Assoc..

[24] Dziewas R., Glahn J., Helfer C., Ickenstein G., Keller J., Ledl C., Lindner-Pfleghar B., Nabavi D.G., Prosiegel M., Riecker A. (2016). Flexible endoscopic evaluation of swallowing (FEES) for neurogenic dysphagia: Training curriculum of the German Society of Neurology and the German stroke society. BMC Med. Educ..

[25] Prikladnicki A., Santana M.G., Cardoso M.C. (2022). Protocols and assessment procedures in fiberoptic endoscopic evaluation of swallowing: An updated systematic review. Braz. J. Otorhinolaryngol..

[26] Onesti E., Schettino I., Gori M.C., Frasca V., Ceccanti M., Cambieri C., Ruoppolo G., Inghilleri M. (2017). Dysphagia in amyotrophic lateral sclerosis: Impact on patient behavior, diet adaptation, and riluzole management. Front. Neurol..

[27] Ballesteros-Pomar M.D., Cherubini A., Keller H., Lam P., Rolland Y., Simmons S.F. (2020). Texture-Modified Diet for Improving the Management of Oropharyngeal Dysphagia in Nursing Home Residents: An Expert Review. J. Nutr. Health Aging.

[28] Baixauli R., Dobiašová A., Tarrega A., Laguna L. (2023). Pairing physical and sensory properties of dysphagia thickeners to understand disliking. Food Hydrocoll. Health.

[29] Vidal-Casariego A., González-Núñez S., Pita-Gutiérrez F., Lugo-Rodríguez G., Martínez-Ramonde T. (2021). Acceptance of different types of thickeners, with and without flavoring, in hospitalized patients with dysphagia—A pilot study. Nutr. Hosp..

[30] Langmore S.E., Pisegna J.M. (2015). Efficacy of exercises to rehabilitate dysphagia: A critique of the literature. Int. J. Speech-Lang. Pathology.

[31] Skidmore E.R., Whyte E.M., Holm M.B., Becker J.T., Butters M.A., Dew M.A., Munin M.C., Lenze E.J. (2010). Cognitive and Affective Predictors of Rehabilitation Participation After Stroke. Arch. Phys. Med. Rehabil..

[32] Howard M.M., Block E.S., Mishreki D., Kim T., Rosario E.R. (2023). The Effect of Sensory Level Versus Motor Level Electrical Stimulation of Pharyngeal Muscles in Acute Stroke Patients with Dysphagia: A Randomized Trial. Dysphagia.

